# HCHs and DDTs in Soils around Guanting Reservoir in Beijing, China: Spatial-Temporal Variation and Countermeasures

**DOI:** 10.1100/2012/628216

**Published:** 2012-12-31

**Authors:** Tie-yu Wang, Bing Tan, Yong-long Lu

**Affiliations:** ^1^State Key Laboratory of Urban and Regional Ecology, Research Center for Eco-Environmental Sciences, Chinese Academy of Sciences, Beijing 10085, China; ^2^College of Environmental Science and Engineering, Liaoning Technical University, Fuxin, Liaoning 123000, China

## Abstract

The concentrations of hexachlorocyclohexanes (HCHs) and dichlorodiphenyltrichloroethanes (DDTs) in the topsoil samples around the Guanting Reservoir in Beijing were measured, and their spatial distribution and environmental risks were analyzed by GIS. The results showed that in 2003, 2007, and 2009, the HCHs concentrations were 0.66, 0.85, and 0.73 ng/g, and the DDTs concentrations were 9.50, 7.80, and 6.46 ng/g in the studied area, respectively. In the topsoil, the HCHs concentrations did not change much while the DDTs concentrations declined steadily. Most of the current residues in soil come from the POPs used in the past years but some new input is also detected in certain regions. The level of HCHs and DDTs residues in the south reservoir is lower than that in the north reservoir. The middle region has the highest HCHs and DDTs concentrations, especially near the Beixinpu town. The high risk regions of pollution of HCHs and DDTs are mainly distributed in the vicinity of Beixinpu town as well. Based on the aforementioned results, a comprehensive countermeasure is proposed entailing decision making, local implementation, scientific support, and public participation with regard to the long-term control and management of POPs around the Guanting Reservoir.

## 1. Introduction

Hexachlorocyclohexanes (HCHs) and dichlorodiphenyltrichloroethanes (DDTs) have been listed as the main pesticidal persistent organic pollutants (POPs) by the United Nations Environment Program (UNEP) in the Stockholm Convention. They have been used in China on a large scale for a long time and the pesticides used in China during 1960–1980 were mainly organochlorine pesticides (OCPs). The consumption of OCPs such as DDTs and HCHs were 192,000 tons in 1970, accounting for 80.1% of the total domestic pesticide production [1, 2]. From 1972 to 1974, the agricultural consumption of HCHs and DDTs amounted to 607.8 tons and 150.8 tons, respectively, in the Beijing municipality [3]. In the early 1980s, the application of pesticidal POPs in the orchards became restricted and after 1983 the comprehensive restrictions on their production and use took effect [4]. However, the environmental problems caused by their toxicity, nonbiodegradability, bioaccumulation, and so forth still seriously threaten human health and environmental well-being [5, 6]. Soil is a reservoir for the OCPs and has gradually changed from a major sink to an important emission source of OCPs to food and drinking water over time. Significant proportions of between 20% and 70% of a pesticide or its degradation products can remain in soil following application [7].

The Guanting Reservoir is located on the Yongding River at 100 km northwest of Beijing. It has a water capacity of 4.2 billion m^3^ and includes 46,000 km^2^ water shed areas. It was previously one of the most important water sources for Beijing until 1997 when its water was polluted. To enable the effective comprehensive management of the Guanting Reservoir and restore its function to supply drinking water, many studies focusing on pesticidal POPs have been carried out around the reservoir [8–10]. However, the potential nonpoint source pollution of POPs from farmlands around the reservoir was rarely reported. Since 2003, we embarked on the continuous integrated and systematic studies by fixing points in the soils around the reservoir [11–16]. Through multiyear monitoring and using GIS technology, we evaluated the pollution situation and risk pattern of pesticidal POPs in the soils around the water shed. The analyses presented in this paper will facilitate better understanding of the residue characteristics and tendency effects of OCPs in soils under various conditions around water sheds. Consequently, we proposed specific countermeasures to remedy regional ecological environment and provided the scientific basis for the comprehensive treatment of the reservoir and a technical framework for water source protection and management projects in the future.

## 2. Materials and Methods

### 2.1. Sample Collection

**Figure 1:**
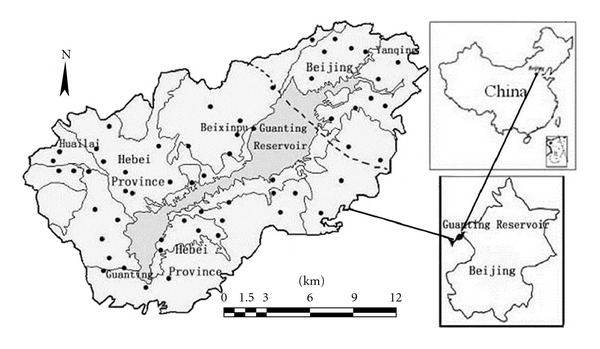
Locations of sampling sites around the Guanting Reservoir.

This study focused on a 920 km^2^ water shed within 2–10 km around the Guanting Reservoir (115.43°E–115.97°E, 40.19°N–40.50°N). The soil types are mainly brown soil, cinnamon soil, and chestnut soil. Land use in the area includes orchards, corn fields, vegetable fields, and forest shrubs. The 58 topsoil samples were collected and analyzed in 2003, 2007, and 2009, respectively. Each sample consisted of a mixture of soil collected from five subsites at a depth of 0–20 cm, each of which covering a radius of 5 m in a cross-pattern. Throughout the survey, a global positioning system (GPS) was used to locate the sampling sites to ensure the valid space registration of the sampling sites in different years. The locations of the sampling sites are shown in Figure 1.

### 2.2. Sample Treatment

All soil samples were air dried at room temperature, then ground with sticks, and passed through a 2 mm nylon mesh to remove grit, plant, and animal residues from the soil samples, and finally stored in polypropylene (PP) plastic bags until analysis. The analysis method follows the previous report [15] and is summarized as follows. Each soil sample (10 g) was weighed accurately and decanted into a 150 mL Erlenmeyer flask, and an appropriate amount of high purity active copper powder and anhydrous sodium sulfate were added to the samples to remove sulfur and water. The sample was then extracted with 100 mL 7 : 3 (v/v) *n*-hexane/dichloromethane in an ultrasonic bath for several times. The extract was transferred into a glass centrifugal tube and centrifuged at 3000 rpm and then decanted into a 250 mL separating funnel where concentrated sulfuric acid was added to purify the extract. After discarding the sulfonated layer, the purified extract was washed at least twice with 10% sodium chloride solution until the pH value of the solution was close to 7 and was then concentrated under reduced pressure to about 2 mL and decanted into a 5 mL graduated test tube. The obtained residue was reduced to a final volume of 0.5 mL under a gentle stream of ultrahigh purity nitrogen (99.99%) for GC analysis.

### 2.3. Sample Analysis

The final extract of pesticidal POPs was analyzed on an HP 6890 gas chromatograph equipped with a ^63^Ni electron capture detector (*μ*ECD) using an HP-1 silica capillary column (30 m × 0.32 mm i.d. × 32 *μ*m). The injector and detector temperatures were 220 and 300°C, respectively. The oven temperature was started from 150°C and increased at 5°C/min, then maintained at 200°C for 2 min, and then increased again at 8°C/min and finally maintained at 270°C for 5 min. The injection was splitless and the injection volume was 1 *μ*L. Ultrahigh purity nitrogen was used as the carrier gas. The Stigma pressure of nitrogen was 60 kPa, and 60 mL/min nitrogen was used as the make-up gas. The pesticidal POPs were identified by retention time and quantified using external standard. 

A mixture of standard solution containing *α*-HCH, *β*-HCH, *γ*-HCH, *δ*-HCH isomers, and pp′-DDE, pp′-DDD, op′-DDT, pp′-DDT were obtained from the National Research Center for Certified Reference Materials of China. For quality assurance and control, procedural blanks and matrixes spiked with the standard solution were analyzed. None of the target compounds were detected in the procedural blanks. All solvents used were distilled in glass (PR grade) and were checked for interferences or contamination prior to use. The amount of substance in the extracts was quantified using the internal standard (2,4,5,6-tetrachloro-*m*-xylene). The recovery of the four HCH isomers (*α*-HCH, *β*-HCH, *γ*-HCH, *δ*-HCH) and four DDT homologues (pp′-DDE, pp′-DDD, op′-DDT, pp′-DDT) were 73.3%, 90.3%, 75.8%, 85.6% and 80.0%, 93.8%, 95.4%, and 96.2%, respectively. The extraction efficiency, as indicated by the recovery of the surrogate standards (TCMX), was 75% ± 10%. The detection limit was set to three times the signal-to-noise ratio (*S*/*N*). The detection limit ranged from 0.06 to 0.15 ng/g for the HCH isomers and from 0.07 to 0.19 ng/g for the DDT homologues. Reagent blanks were also analyzed simultaneously with the experimental samples.

### 2.4. Data Processing

The software used for the mapping and spatial analysis was ArcGis 10.0(ESRI, US). An interpolation method called Ordinary Kriging was adopted for the interpolation of geographical data. SPSS 11.5 for Windows was employed for statistical analysis. Excel (version 2007, Microsoft) was also used for preliminary data analysis and histogram graphing.

## 3. Results and Discussion

### 3.1. Temporal Variation of Residual Pesticidal POPs in Soils

Table 1 shows that, from 2003 to 2009, the HCHs concentrations in topsoil did not change much, while the DDTs concentrations declined steadily. Specifically, the *β*-HCH isomers were enriched in the soil, whereas the content of *α*-HCH isomers declined. The concentrations of *γ*-HCH isomer (0.01 ng/g dw) and *δ*-HCH isomer (0.02 ng/g dw) in 2009 are also less than those in 2003 (0.06 ng/g dw and 0.19 ng/g dw) or 2007 (0.16 ng/g dw and 0.07 ng/g dw). The ratio of *α*-HCH to *γ*-HCH is often considered the characteristic index to determine the source of the HCHs. The value of ratio between 4 and 7 in samples implies that there may be new source of HCHs; a ratio close to 1 may indicate new industrial input of lindane and a ratio greater than 7 may suggest long distance atmospheric transport of HCHs [17]. As Table 2 shows 8.9% of the samples in 2003 and 15% of the samples in 2007 had *α*-HCH/*γ*-HCH ratio less than 1. The result indicates that the main HCH input is from lindane and there may have been new industrial pollution source after restricting HCH usage. Previous studies by Wang et al. (2003), Sun et al. (2005), and Xue et al. (2005) on the Guanting Reservoir also showed that the HCHs in water may be due to the input of lindane or industrial HCHs from the upstream or surrounding regions [3, 8, 9]. However, according to the environmental monitoring in 2009, there was no new industrial lindane pollution source near the Guanting region, because *α*-HCH/*γ*-HCH ratio of each sample was much higher than 1. 

**Figure 2:**
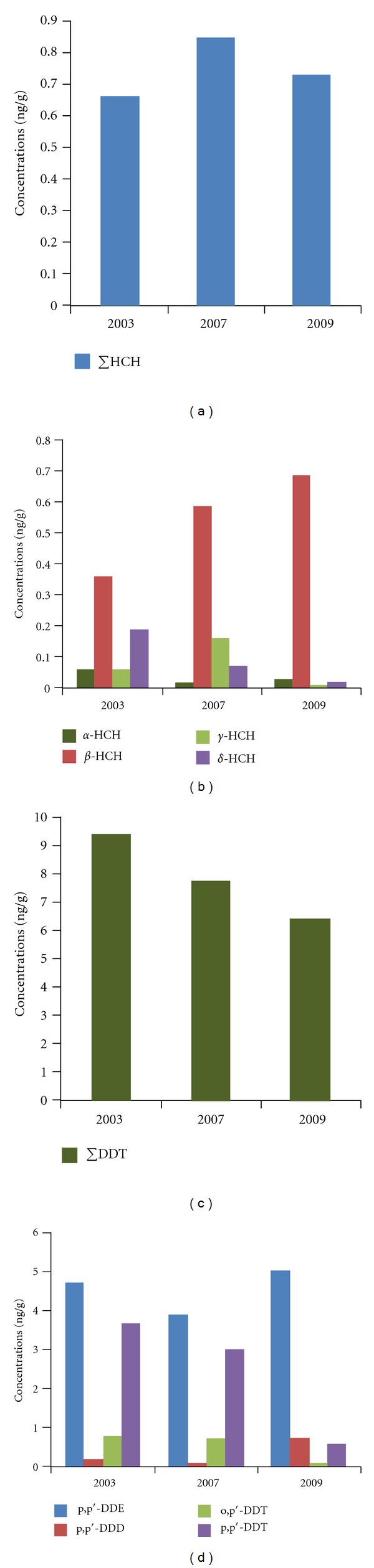
Temporal trends of HCHs and DDTs in soils from 2003 to 2009.

By comparing with previously reported results [16, 18, 19], it can be seen that DDTs have always been the main components of pesticidal POPs in the Guanting Reservoir area, that is, 93.2% in 2003, 90.2% in 2007, and 90.6% in 2009, respectively.  Figure 2 shows that the concentration of pp′-DDE in soil is the greatest in 2009. Besides, the concentration of pp′-DDT in soil gradually decreased from 2003 to 2009 while the concentration of pp′-DDD gradually increased. These results indicate that pp′-DDT has gradually degraded to pp′-DDD and pp′-DDE mainly through aerobic degradation. Although DDT can degrade relatively more easily, its degradation products DDE and DDD are more stable and more difficult to degrade. The ratio of (DDE + DDD) to pp′-DDT is usually considered a characteristic index to determine the source of DDT in the environment [20]. Table 2 shows that 23.2% of the samples in 2003 and 41.7% of the samples in 2007 had a (DDE + DDD) to pp′-DDT ratio of less than 1. This indicates that DDTs such as dicofol or other pesticides containing DDTs as by-product were still used during that time in this region. Dicofol products have a high DDT content varying from 3.54% to 10.8%. Since the 1960 s, in each year 2000 tons of dicofol products were put into use. As a result, dicofol may be the new source of DDT pollution [21]. Table 2 also shows that only about 8.2% of the samples had a (DDE + DDD) to pp′-DDT ratio less than 1 in 2009. Hence, this new DDT source is basically under control and the environmental exposure was mainly due to the historical use of dicofol.

Concentrations of HCHs and DDTs in soils were compared with the soil quality reference values recommended in China and The Netherlands [22]. According to the guidelines of the Chinese environmental quality standards for soil (GB 15618-1995), the limits for HCH and DDT in the soils are both 50, 500, and 1,000 ng/g dw, corresponding to Class I, II, and III, respectively. In 2003 and 2007, concentrations of HCHs in all soil samples were below the limit of Class I (50 ng/g dw) and Dutch target value (10 ng/g dw). While in 2009, HCHs concentrations were also within the limit of Class I but in several sites exceeded the Dutch target value. With regard to DDTs, mean concentrations of DDTs were within the criteria for Class I and Dutch target value in different years. In general, even concentrations of HCHs and DDTs were both less than the Chinese criteria Class I, which is set to ensure the safety of agricultural products and to prevent foods contamination from HCHs and DDTs; they still could have negative impacts on local atmosphere and aquatic ecosystem in the long term because of their nonbiodegradability and bioaccumulation. 

### 3.2. Spatial Variation of Pesticidal POPs in Soils

**Figure 3:**
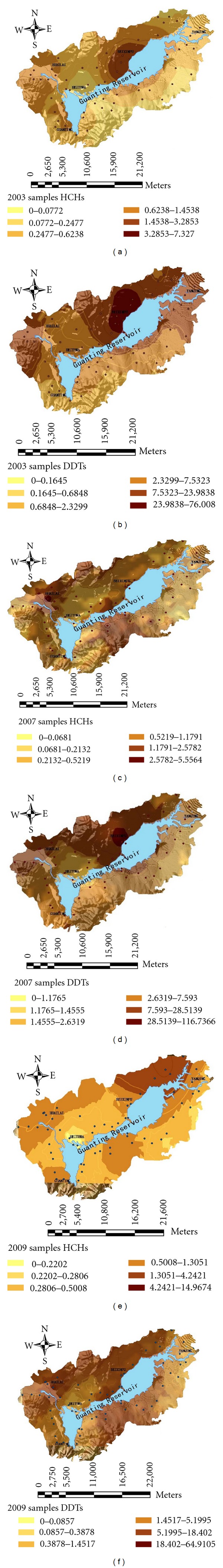
Spatial-temporal trend of HCHs and DDTs in soils around the Guanting Reservoir.

The spatial evolution patterns of HCHs and DDTs in soils were analyzed using the Geostatistical Analyst in the GS extension module of ArcGis with ordinary Kriging interpolation. The results in Figure 3 show that, in 2003, 2007, and 2009, the residual pesticidal POPs vary significantly in different areas around the Guanting Reservoir. The HCHs and DDTs are mainly concentrated in the Beixinpu town and the Huailai county, and their concentrations decreased from north to south. Generally speaking, the concentrations of HCHs and DDTs in the south reservoir were lower than those in north reservoir both before and after the pesticide restriction took effect. This is because (1) agriculture is less developed in the south reservoir and (2) the sandy and weathering soils in the south reservoir speed up the degradation and evaporation of POPs. The Beixinpu town is located in the middle of the north reservoir area. It connects the mountains in the north with the water in the south and has a decreasing geomorphological gradient from north to south. The Beixinpu town has large areas of shrub forests and orchards and used large amounts of HCH and DDT in the 1970s for forestry pest control. Currently lots of pesticides that may contain POPs isomers or degradation products (such as dicofol) are still consumed in the orchards in this area. This may explain why the residual amounts of HCH and DDT were high in the Beixinpu town compared with other areas.

The Huailai county (in Hebei province) located in the central and western Guanting Reservoir area was found to have high HCHs exposures, and the Beixinpu town was found to have the highest risk. The concentration of HCHs in the Beixinpu town was relatively high in 2003, but, after six years of degradation, it decreased to a normal level not significantly different with its surrounding. Previous studies have also shown that the concentrations of heavy metals in the Beixinpu town are also very high [13, 23]. The Yanghe river and the Sanggan river are the major pollution sources of the Guanting Reservoir. Although the insecticide factories, chemical plants, and printing and dyeing mills located in Zhangjiakou and Xuanhua at the upstream of the Yanghe river have been partially shut down, serious pollution has already occurred. Furthermore, because of the agricultural irrigation in those water sheds, the pollutants including pesticidal POPs were spread to the surrounding soils, leading to high environmental risks. In 2003, the soil DDTs were mainly concentrated in three regions: the western reservoir (near Huailai county), the central reservoir (Beixinpu town), and the eastern reservoir (Yanqing county). The DDT concentrations in the northern regions were higher than those in the southern regions (Figure 3). In particular, the large historical use of DDTs in the Beixinpu area and the new input of pesticidal POPs resulted in much higher DDTs residues in those regions compared with their surroundings, which shows a patchy distribution of high pollution risks. Until 2007, the high pollution risk areas were still concentrated in these three regions, but the acreage of the high pollution risk areas decreased in the central Beixinpu town and Yanqing county. In 2007 the soil DDTs showed no obvious regional aggregation and its overall level declined substantially. Until 2009, the concentrations of DDTs in the high pollution risk areas in the northern Guanting Reservoir further declined from a maximum of 116.74 ng/g dw to 64.91 ng/g dw (Table 1). According to the Chinese Environmental Quality Standard for soils, the concentrations of HCHs in this area were far below the first grade (class I = 50 ng/g dw). Only two sampling sites had DDTs concentrations slightly exceeding the first grade in 2009 (64.91 ng/g dw and 59.05 ng/g dw, resp.), which accounted for only 3.3% of all samples. Overall, after years of pesticide restriction and natural degradation, the pesticidal POPs residues in the soils of studied areas decreased steadily. However, new pollution sources such as lindane and dicofol may exist in some sampling sites and should receive more attention.

### 3.3. Management and Control of Pesticidal POPs around the Guanting Reservoir

#### 3.3.1. Integrated Management Framework for Regional POPs Control

**Figure 4:**
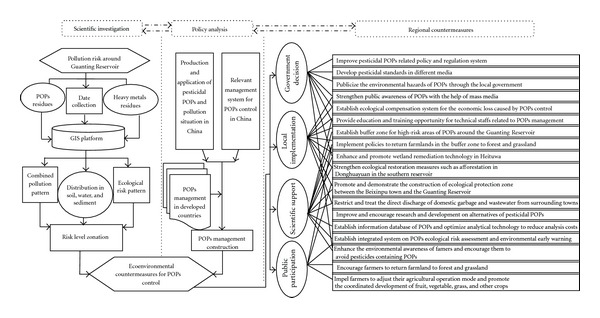
Framework of regional POPs management in the Guanting area.

By analyzing the temporal and spatial distribution pattern of pesticidal POPs around the Guanting Reservoir based on previous and present studies, a framework of ecoenvironmental countermeasures is put forward for regional POPs control (Figure 4) [24–26]. The establishment and implementation of regional policies should be based on scientific investigation, led by policy analysis, and completed under the common goal of governmental decision-making, local implementation, scientific support, and public participation. This framework includes 18 specific regional eco-environmental countermeasures (1) to improve the policy and the regulation system related to pesticidal POPs; (2) improve or develop pesticidal standards and criteria in different media; (3) publicize the environmental hazards of POPs through the local government; (4) strengthen public awareness of POPs through mass media; (5) establish the ecological compensation system for the economic loss due to POPs control; (6) provide education and training opportunity for the technical staffs of POPs management; (7) establish buffer zones around the Guanting reservoir for high-risk areas of POPs; (8) implement policies to return farmlands in the buffer zone to forest and grassland; (9) enhance the diffuse wetland remediation technology in Heituwa; (10) strengthen the ecological restoration measures such as afforestation in Donghuayuan in the southern reservoir; (11) promote and demonstrate the construction of ecological protection zone between the Beixinpu town and the reservoir; (12) restrict and control the direct discharge of domestic garbage and wastewater from surrounding towns; (13) improve and encourage R&D on alternatives of pesticidal POPs; (14) establish the POPs information database and optimize the analytical technology for POPs; (15) establish integrated system on ecological risk assessment and environmental earlywarning of POPs; (16) enhance the environmental awareness of farmers and encourage them to avoid pesticides containing POPs; (17) encourage farmers to return farmland to forest and grassland; (18) impel farmers to adjust agricultural operation mode and promote the coordinated development of fruit, vegetable, grass, and other crops.

#### 3.3.2. Ecoengineering Measures to Control POPs in Specific Areas

**Figure 5:**
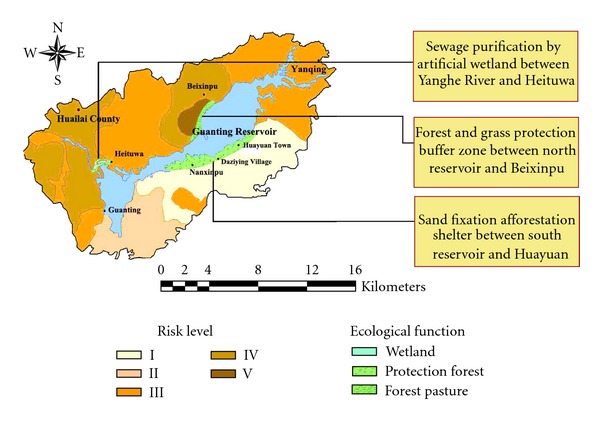
Ecological engineering projects around the Guanting Reservoir.

With the use of overlay analysis in GIS, the ecological risk near the Guanting Reservoir was classified into five grades based on its spatial distribution pattern. Meanwhile, specific ecological countermeasures for typical regions are proposed in Figure 5.

**Sewage Purification by Artificial Wetland between Yanghe River and Heituwa**

The chemical plants located at the upstream of the Yanghe river in Zhangjiakou once caused serious pollution in the Guanting Reservoir. The Yanghe river lies between the reservoir in the Yongding river and the Huailai county and still has high concentrations of HCHs and DDTs. Therefore, to protect the water quality of the Guanting Reservoir, the environmental management and ecological restoration in this region must be strengthened. The construction of the Heituwa artificial wetland is a crucial part of the water quality improvement project of the Guanting Reservoir, and it is also the first ecological barrier to the polluted water from Yongding river flowing into the Guanting Reservoir. The construction of 84 hectares of ecological purification ponds and 7.4 hectares of wetland has been planned in the Heituwa artificial wetland. A floating-leaved plant zone of 2.52 hectares and an emergent vegetation zone of 6.84 hectares will be artificially planted, leaving the remaining areas to be covered by natural reproduction.

For ecological purification, the artificial wetland construction could also be carried out in some other areas based on the experience of the Heituwa artificial wetland. The construction of another artificial wetland at the upstream of the Guishui river near the Yanqing county is recommended to effectively prevent the pollution from industrial activities and domestic sewage discharge. The consolidated utilization of forests, arbor, shrubs, and grass can purify surface runoff, reduce reservoir sedimentation, alleviate nonpoint source pollution, recover the ecological functions, and improve the landscape around reservoir.

**Forest and Grass Protection Buffer Zone between North Reservoir and Beixinpu**

The large areas of orchards in the Beixinpu town are a substantial source of pesticides and fertilizers. It was reported that the Beixinpu town was once the most heavily polluted region around the Guanting Reservoir, in particular suffering from combined pollutions of DDTs and HCHs [23]. A buffer zone is strongly recommended in the sensitive areas around the reservoir between the Beixinpu town and the Huailai county. Through returning farmland to forest and grassland, the construction of a buffer strip approximately 7 km long and 200 m wide is recommended between the orchards and the water body. This buffer system can successively filtrate the pollutants through farm (orchard), forestry, shrub (grass), and water, which not only blocks the input of pesticidal POPs from the Beixinpu town due to runoffs, infiltration, and soil erosion but also fixes those POPs coming from short-distance atmospheric migration or pesticides-cemented dusts.

**Sand Fixation Afforestation Shelter between South Reservoir and Huayuan**

The south reservoir has poor sandy soils and is not suitable for agriculture, forestry or grass cultivation. The construction of a shelter forest approximately 14 km long and 500 m wide is proposed, using shrubs with barren resistance and low water consumption.

Meanwhile, the corresponding economic compensation system should be established for the farmers affected by the ecological protection and agriculture limitation policies. In order to transform the POPs management from passive control to active prevention, it is necessary to strengthen the public awareness of the environmental and health hazards of POPs, promote relevant policies and regulations, and foster public understanding and coordination.

## 4. Conclusions

This study measured the spatial and temporal variability of HCH and DDT concentrations in the soils around the Guanting Reservoir of Beijing. From 2003 to 2009, the DDT concentrations decreased significantly while the HCH concentrations did not change much. The agricultural pesticide application in this region at earlier times was the main source of HCH and DDT. In the past several decades, China has produced and used large quantities of pesticidal POPs for agriculture purposes and for preventing infectious diseases. Therefore, monitoring databases, especially long-term systematic survey, should be expanded to fully understand this problem.

On the basis of previous and present studies on pesticidal POPs around this water shed, an integrated regional POPs control framework was established. The framework involves scientific investigation, policy analysis, and regional countermeasures, and 18 specific countermeasures are proposed that cover government decision making, local implementation, scientific and technology support, as well as public participation.

## Figures and Tables

**Table 1:** Concentrations (ng/g dw) of OCPs in soil samples in 2003, 2007, and 2009.

Variables		Mean ± SD (min–max)		
		2003	2007	2009
HCHs group	*α*-HCH	0.06 ± 0.14 (nd−0.78)	0.02 ± 0.05 (nd−0.27)	0.03 ± 0.08 (nd−0.48)
	*β*-HCH	0.36 ± 0.68 (nd−2.74)	0.59 ± 0.94 (nd−4.58)	0.69 ± 1.83 (nd−14.34)
	*γ*-HCH	0.06 ± 0.20 (nd−1.41)	0.16 ± 0.19 (nd−1.01)	0.01 ± 0.03 (nd−0.15)
	*δ*-HCH	0.19 ± 0.61 (nd−3.55)	0.07 ± 0.22 (nd−1.53)	0.02 ± 0.06 (nd−0.30)
	∑HCH	0.66 ± 1.34 (nd−7.33)	0.85 ± 1.18 (nd−5.56)	0.73 ± 1.92 (nd−14.97)
DDTs group	p,p′-DDE	4.71 ± 9.42 (nd−52.20)	3.88 ± 10.69 (nd−78.07)	5.07 ± 11.59 (nd−55.96)
	p,p′-DDD	0.24 ± 0.61 (nd−3.37)	0.11 ± 0.33 (nd−1.76)	0.73 ± 1.70 (nd−9.71)
	o,p′-DDT	0.84 ± 2.30 (nd−11.71)	0.75 ± 1.81 (nd−9.04)	0.08 ± 0.47 (nd−3.55)
	p,p′-DDT	3.67 ± 7.75 (nd−33.08)	2.98 ± 7.38 (nd−50.73)	0.58 ± 1.30 (nd−7.34)
	∑DDT	9.46 ± 17.76 (nd−76.01)	7.84 ± 17.68 (nd−116.74)	6.46 ± 13.28 (nd−64.91)
Reference		[16]	[19]	Present study

*nd: the value less than LOD (limits of detection) was set to nd.

**Table 2:** Composition of HCH and DDT in soil samples in 2003, 2007, and 2009.

Ratio	Year	Average	Minimum	Maximum	Percentage of sites with a ratio less than 1
*α*-HCH/*γ*-HCH	2003	1.35	0.25	3.35	8.9%
	2007	0.42	0.12	0.73	15.0%
	2009	2.76	2.25	3.26	0

(DDE + DDD)/p,p′-DDT	2003	2.52	0.24	25.2	23.2%
	2007	1.25	0.09	4.22	41.7%
	2009	7.07	0.24	40.1	8.2%
